# Constructing recombinant *Saccharomyces cerevisiae* strains for malic-to-fumaric acid conversion

**DOI:** 10.1093/femsle/fnad003

**Published:** 2023-01-16

**Authors:** Annica Steyn, Marinda Viljoen-Bloom, Willem Heber Van Zyl

**Affiliations:** Department of Microbiology, Stellenbosch University, Stellenbosch 7600, South Africa; Department of Microbiology, Stellenbosch University, Stellenbosch 7600, South Africa; Department of Microbiology, Stellenbosch University, Stellenbosch 7600, South Africa

**Keywords:** fumarase, fumaric acid, malic acid, malate transporter, *Saccharomyces cerevisiae*, organic acids

## Abstract

*Saccharomyces cerevisiae* with its robustness and good acid tolerance, is an attractive candidate for use in various industries, including waste-based biorefineries where a high-value organic acid is produced, such as fumaric acid could be beneficial. However, this yeast is not a natural producer of dicarboxylic acids, and genetic engineering of *S. cerevisiae* strains is required to achieve this outcome. Disruption of the natural *FUM1* gene and the recombinant expression of fumarase and malate transporter genes improved the malic acid-to-fumaric acid conversion by engineered *S. cerevisiae* strains. The efficacy of the strains was significantly influenced by the source of the fumarase gene (yeast versus bacterial), the presence of the XYNSEC signal secretion signal and the available oxygen in synthetic media cultivations. The *ΔFUM1Ckr_fum* + *mae1* and *ΔFUM1*(ss)*Ckr_fum* + *mae1* strains converted extracellular malic acid into 0.98 and 1.11 g/L fumaric acid under aerobic conditions.

## Teaser

Engineering baker’s yeast for malic-to-fumaric acid conversion through overexpression of fungal fumarase and malate transporter genes.

## Introduction

Various four- and six-carbon dicarboxylic acids, including succinic and fumaric acid, have been included in the ‘Top Value-Added Chemicals from Biomass’ list compiled in the early 2000s (Werpy and Petersen 2004). Their applications in various important industries and global market values explain the substantial interest in the microbial production of these dicarboxylic acids from relatively cheap substrates (Yin et al. 2015, Njokweni et al. 2021, Xu et al. 2022). Fumaric acid is traditionally produced via three routes (Xu et al. 2012). The petrochemical route involves maleic anhydride conversion with high yields, but this is becoming more expensive due to increased oil prices (Ichikawa et al. 2003). Chemical synthesis of fumaric acid can have a negative environmental impact as it requires high pressures and temperatures as well as heavy metal catalysts and organic solvents (Raab et al. 2010). Lastly, the fermentative route has mainly been based on fungal fermentations with *Rhizopus* species (such as *Rhizopus oryzae* and *Rhizopus arrhizus*) (Engel et al. 2008), but the cell morphology and growth challenges of these fungi limit their use on industrial scale. There is also concern about product safety due to the potential pathogenic properties of some of these fungi (Xu et al. 2012).

The high market price and limited availability of maleic anhydride, the precursor for fumaric acid, highlight the need for alternative strategies to produce fumaric acid (ChemAnalyst 2022). One such strategy is to produce fumaric acid from a renewable feedstock using microbial strains in a biorefinery setup from a malic-acid-rich substrate (e.g. apple or grape pomace). Potential substrates include renewable biomass and agricultural waste that could provide for a mixture of high- and low-value products, such as fumaric acid and ethanol. The yeast *Saccharomyces cerevisiae* is an ideal candidate for the bio-fermentative production of dicarboxylic acids, including fumaric acid (Xu et al. 2012, 2022). It is a well-established industrial microorganism in the food and beverage industry and is tolerant towards inhibitors, acidic conditions, and high sugar and ethanol concentrations (Nevoigt 2008). However, the yeast’s native fumarases predominantly catalyze the unidirectional conversion of fumaric acid to malic acid (Pines et al. 1996, Ilica et al. 2019). The recombinant expression of appropriate genes is thus required to direct malic acid degradation towards the production of fumaric acid in *S. cerevisiae* (Steyn et al. 2021).

Previous studies explored the genetic modification of *S. cerevisiae* strains to produce fumaric acid, often via the conventional glycolytic and reductive tricarboxylic acid (TCA) pathways and overexpressing target genes, such as *FUM1, MDH* and *PYC* from *R. oryzae* (Xu et al. 2012, 2013, Chen et al. 2016, Guo et al. 2020). Endogenous yeast fumarases mainly catalyze the unidirectional conversion of fumaric acid to malic acid (Pines et al. 1996, Ilica et al. 2019). Since this inhibits the potential accumulation of fumaric acid in the yeast cytosol, deletion of the natural *S. cerevisiae* fumarase gene resulted in higher fumaric acid yields and titres (Xu et al. 2013, Wei et al. 2015, Chen et al. 2016).

The expression of a recombinant transporter that allows for active transport of l-malate and other C_4_-dicarboxylic acids (Saayman and Viljoen-Bloom 2006) could advance fumaric acid production. The very low transport rate of malic acid in yeasts such as *S. cerevisiae* mainly relies on passive diffusion (Volschenk et al. 2003), whereas *Schizosaccharomyces pombe* cells are equipped with a natural malate transporter (SpMae1) (Grobler et al. 1995). When this transporter is expressed in *S. cerevisiae*, the strains can mediate malate import and export (Volschenk et al. 1997b, Camarasa et al. 2001). Furthermore, the constitutive proton-dicarboxylate symport system of *S. pombe* is not subject to glucose repression and functions well in the presence of high glucose concentrations (Sousa et al. 1992). A study on the transport of dicarboxylic acids in *S. cerevisiae* suggested that the *S. pombe* transporter (SpMae1p) may belong to a voltage-gated anion channel family (SLAC1) (Darbani et al. 2019), rather than being a member of the tellurite-resistance/dicarboxylate transporter (TDT) family that uses a proton motive force (Grobler et al. 1995). The SpMae1p displayed high activity towards C4-dicarboxylic acids (e.g. fumaric, malic, and succinic acids) without inhibiting cell growth at low or neutral pH values (Darbani et al. 2019).

In this study, recombinant *S. cerevisiae* strains were constructed to produce fumaric acid from malic-acid-containing sources using an exogenous fumarase and transport system. While most previous studies focussed on fumarase genes from *Rhizopus* species, the current study employed yeast (*Candida kruseii*) and bacterial (*Escherichia coli*) fumarase genes. These genes were expressed with/without the *S. pombe mae1* transporter gene, which reportedly enhanced fumaric acid production when expressed in recombinant *S. cerevisiae* strains (Zelle et al. 2008). We also investigated whether adding the XYNSEC secretion signal from *Trichoderma reesei* xylanase 2 (Njokweni et al. 2012) could enable extracellular malic-to-fumaric acid conversion. All the yeast strains were evaluated on different carbon sources in small- and upscaled cultivations using defined media.

## Materials and methods

### Strains, plasmids, genes, and primers

The yeast strains used in this study were derived from two auxotrophic *S. cerevisiae* strains, Y11030 (EUROSCARF, Oberursel, Germany) and the laboratory strain Y294 (ATCC 201160). The Y11030 strain (derived from *S. cerevisiae* S288c) has a disrupted fumarase (*FUM1*) gene on chromosome 16, referred to as Sc-FUM1⁻. The details of the strains and plasmids used in this study are listed in Table 1.

**Table 1.** Strains and plasmids used in this study.

Strains/Plasmids	Genotype	Source
Strains:		
Sc-FUM1^−^ (*S. cerevisiae* Y11030)	*BY4742, MATα, ura3Δ0, leu2Δ0, his3Δ1, lys2Δ0, YPL262w::kanMX4*	EUROSCARF
*S. cerevisiae* Y294	*MATalpha, leu23, leu2112, ura352, his3deltatrp1 GAL*+ [*cir*+]	ATCC 201160
*Escherichia coli* DH5α	*supE44, ΔlacU169*, (*φ80lacZΔ*M15), *hdR17, recA1, endA1, gyrA96, thi-1, relA1*	Sambrook et al. (1989)
*ΔFUM1Ckr_fum*	Sc-FUM1^−^ transformed with pBBH1-Ckr_fum	This study
*ΔFUM1Ckr_fum* + *mae1*	Sc-FUM1^−^ transformed with pBBH1-Ckr_fum and pHV3	This study
*ΔFUM1Ckr_fum* + *LEU2*	Sc-FUM1^−^ transformed with pBBH1-Ckr_fum and YEplac181	This study
*ΔFUM1*(ss)*Ckr_fum*	Sc-FUM1^−^ transformed with pBBH4-Ckr_fum	This study
*ΔFUM1*(ss)*Ckr_fum* + *mae1*	Sc-FUM1^−^ transformed with pBBH4-Ckr_fum and pHV3	This study
*ΔFUM1*(ss)*Ckr_fum* + *LEU2*	Sc-FUM1^−^ transformed with pBBH4-Ckr_fum and YEplac181	This study
*ΔFUM1*(ss)*Eco_fum*	Sc-FUM1^−^ transformed with pBBH4-Eco_fum	This study
*ΔFUM1*(ss)*Eco_fum* +* mae1*	Sc-FUM1^−^ transformed with pBBH4-Eco_fum and pHV3	This study
*ΔFUM1*(ss)*Eco_fum* +* LEU2*	Sc-FUM1^−^ transformed with pBBH4-Eco_fum and YEplac181	This study
WT*Ckr_fum*	Y294 transformed with pBBH1-Ckr_fum	This study
WT*Ckr_fum* +* mae1*	Y294 transformed with pBBH1-Ckr_fum and pHV3	This study
WT*Ckr_fum* +* LEU2*	Y294 transformed with pBBH1-Ckr_fum and YEplac181	This study
WT(ss)*Ckr_fum*	Y294 transformed with pBBH4-Ckr_fum	This study
WT(ss)*Ckr_fum* +* mae1*	Y294 transformed with pBBH4-Ckr_fum and pHV3	This study
WT(ss)*Ckr_fum* +* LEU2*	Y294 transformed with pBBH4-Ckr_fum and YEplac181	This study
WT(ss)*Eco_fum*	Y294 transformed with pBBH4-Eco_fum	This study
WT(ss)*Eco_fum* +* mae1*	Y294 transformed with pBBH4-Eco_fum and pHV3	This study
WT(ss)*Eco_fum* +* LEU2*	Y294 transformed with pBBH4-Eco_fum and YEplac181	This study
Plasmids:		
pUC57-Ckr_fum	pUC57 carrying synthetic *Ckr_fum*	This study
pUC57-Eco_fum	pUC57 carrying synthetic *Eco_fum*	This study
pBBH1	*bla, URA3, ENO1_P_-ENO1_T_*	Njokweni et al. (2012)
pBBH1-Ckr_fum	*bla, URA3, ENO1_P_-Ckr_fum-ENO1_T_*	This study
pBBH4	*bla, URA3, ENO1_P_-XYNSEC-ENO1_T_*	Nevoigt (2008)
pBBH4-Ckr_fum	*bla, URA3, ENO1_P_-XYNSEC-Ckr_fum-ENO1_T_*	This study
pBBH4-Eco_fum	*bla, URA3, ENO1_P_-XYNSEC-Eco_fum-ENO1_T_*	This study
YEplac181	*LEU2*	Gietz and Sugino (1988)
pHV3	YEplac181, *LEU2, PGK1_P_-mae1-PGK1_T_*	Volschenk et al. (1997a)

The yeast episomal plasmids, pBBH1 and pBBH4, were used for recombinant expression of the fumarase genes. Both plasmids contain the *URA3* marker and the *ENO1* promoter and terminator sequences, whilst pBBH4 includes the *T. reesei* XYNSEC secretion signal upstream of the cloning site (Njokweni et al. 2012). Plasmid pHV3 (Volschenk et al. 1997a), containing the *S. pombe* malate permease gene (*mae1*) and *LEU2* selectable marker, was used to promote the active transport for malic acid and fumaric acid, as opposed to passive diffusion. Plasmid YEplac181 served as a *LEU2* control.

The fumarase gene (*fumC*) (fumarate hydratase; GenBank accession number ATL11923.1; Finley et al. 2013) from *Issatcheckia orientalis* [now classified as *Pichia kudriavzevii* (teleomorph) or *C. kruseii* (anamorph)] was selected based on its previous inclusion in the BioAmber Inc. strains for succinic acid production and reports that its overexpression increased fumaric acid production three-fold (Gu et al. 2018). To remove an internal *Bgl*II restriction site in the *fumC* DNA sequence, the AGA nucleotides at position 139–141 were replaced with AGG to retain the arginine codon, creating the *Ckr_fum* gene. The *E. coli fumC* DNA sequence (fumarate hydratase; GenBank accession number KGA86907.1) was codon-optimized for expression in *S. cerevisiae* and is referred to as *Eco_fum*. The synthetic fumarase genes (GenScript, Piscataway, NJ, USA) include 5′ *Eco*RI and 3′ *Xho*I restriction sites for subcloning in pUC57. The predicted amino acid sequences were analyzed with SignalP-5.0 software to confirm that neither had a secretion signal peptide.

Yeast-Mediated Ligation (YML) primer sets were designed for amplification and detection of the *Ckr_fum* and *Eco_fum* genes (Table 2), and primers F-FUM1(63 U) and R-FUM1(86D) to confirm disruption of *FUM1* in Sc-FUM1⁻. Primer set J13–14 (Husnik et al. 2006) was used to confirm the presence of the *mae1* gene with colony PCR. All primers were synthesized by Inqaba Biotechnical Industries (Pretoria, South Africa).

**Table 2.** PCR primers designed for gene amplification (restriction sites are underlined).

Primer name	Sequence (5′–3′)
F-ENO1_P_-*Eco*RI-M-Ckr_fum	GCTTATCAACACACAAACACTAAATCAAAGAATTCATGTTAGCTGCTAGATCATTAAAG
R-ENO1_T_-*Xho*I-(S)-Ckr_fum	GCTTAATCAAAAGCTCTCGAGTTAATCCTTTGGACCAATCATGTTTTCTGG
F-XYNSEC-*Nru*I-M-Eco_fum	CCCGTGGCTGTGGAGAAGCGCTCGCGAATGAACACTGTTAGATCAGAAAAGG
R-ENO_T_-*Xho*I-(S)-Eco_fum	GGACTAGAAGGCTTAATCAAAAGCTCTCGAGTTATCTACCAGCTTTCATAGAACCAACC
F-FUM1(63 U)	CGTGACTTTTAGTACTGCAGCTG
R-FUM1(86D)	CCTTAGCGGAGGGACCATTG
F-J13^a^	AACCAAAAATGGTACCAAGCTTTCTAACTGATCTATCCAAAACTGA
R-J14^a^	AAGGAAAAAAGGTACCAAGCTTTAACGCAGAATTTCGAGTT

a Husnik et al. (2006).

### Strain construction

#### Confirmation of *FUM1* disruption in Sc-FUM1⁻

Disruption of the *FUM1* gene in strain *S. cerevisiae* Sc-FUM1⁻ was confirmed via PCR using the F-FUM1(63U) and R-FUM1(86D) primers. Genomic DNA was extracted (Laemmli 1970) from the Sc-FUM1⁻ and *S. cerevisiae* S288c (control) strains, and 5 μL genomic DNA was added to a 25-μL PCR reaction mixture containing 10× Standard *Taq* Reaction Buffer (Dream*Taq*) (Thermo Fisher Scientific, Waltham, MA, USA), *Taq* polymerase, 10 mM dNTPs, and 10 μM of each primer. Gene amplification was performed with an Applied Biosystems 2720 thermal cycler (Applera Corporation, MA, USA) with an initial denaturation step at 95°C for 5 min, followed by 35 cycles of denaturation at 95°C for 30 s, annealing at 55°C for 30 s, extension at 72°C for 1 min, and a final extension at 72°C for 7 min.

PCR products were separated and visualized with 1% agarose gel electrophoresis (SeaKem® LE, Lonza, Basel, Switzerland) and purified with the Nucleofast 96-well post-PCR clean-up plate (Macherey-Nagel, Düren, Germany) on a Tecan EVO150 robotic workstation at Stellenbosch University’s Central Analytical Facilities (CAF). The nucleotide sequence of the products was determined with the BigDye Terminator V3.1 sequencing kit (Applied Biosystems) and adjusted within Chromas Lite version 2.01 (Technelysium, Queensland, Australia) for alignment and comparison to the *S. cerevisiae* S288c (control) *kanMX* marker sequence (http://www.ncbi.nlm.nih.gov/blast).

#### Cloning and subcloning of fumarase genes

The two synthetic fumarase genes were cloned in the 5′ *Eco*RI and 3′ *Xho*I restriction sites of pUC57 and transformed into competent cells of *E. coli* DH5α (Sambrook et al. 1989). Transformants were selected on Luria–Bertani agar plates (5 g/L yeast extract, 10 g/L tryptone, 10 g/L NaCl, and 20 g/L bacteriological agar) (Sigma–Aldrich, Steinheim, Germany), supplemented with 100 mg/mL ampicillin (Roche Diagnostics, Basel, Switzerland), and incubated at 37°C. The small-scale cetyltrimethylammonium bromide method (Del Sal et al. 1988) was used to extract plasmids and the DNA concentrations were quantified with the BioDrop DUO UV/VIS Spectrophotometer (Biochrom, Cambridge, UK).

The *Ckr_fum* and *Eco_fum* genes were amplified from their respective pUC57 plasmids using the primer sets F-ENO1_P_-EcoRI-M-Ckr_fum + R-ENO1_T_-XhoI-(S)-Ckr_fum and F-XYNSEC-NruI-M-Eco_fum + R-ENO_T_-XhoI-(S)-Eco_fum, respectively. The 25-μL PCR reaction mixtures were set up as described above with 1 μL plasmid DNA with an initial denaturation step at 95°C for 5 min, followed by 25 cycles of denaturation at 95°C for 30 s, annealing at 60°C for 30 s, extension at 72°C for 1 min, and a final extension at 72°C for 7 min.

The *Ckr_fum* and *Eco_fum* genes were subcloned into the pBBH1 and pBBH4 expression plasmids using YML; pBBH4 carries the XYNSEC signal for extracellular secretion of recombinant proteins in *S. cerevisiae* (Njokweni et al. 2012). The pBBH1 vector was linearized with *Eco*RI and *Xho*I and pBBH4 with *Nru*I and *Xho*I (Inqaba Biotechnical Industries). A NucleoSpin Gel and PCR Clean–up kit (Macherey-Nagel) was used to extract and purify the DNA fragments from the agarose gel, and the DNA concentrations were determined with a BioDrop DUO UV/VIS Spectrophotometer.

YML and electroporation (Sambrook et al. 1989, Cripwell et al. 2019) were used to clone the amplified *Ckr_fum* and *Eco_fum* genes into the linearized pBBH1 and pBBH4 vectors. The Sc-FUM1⁻ and *S. cerevisiae* Y294 strains were cultivated to saturation in YPD broth (20 g/L glucose, 10 g/L yeast extract, and 20 g/L peptone; pH 5.5) and prepared for electroporation. The transformed cells were spread onto SC⁻^URA^ plates [Synthetic Complete medium: 1.7 g/L yeast nitrogen base without amino acids and ammonium sulphate (BD-DIFCO™, NJ, USA), 20 g/L glucose, 5 g/L ammonium sulphate, 1.5 g/L yeast synthetic drop-out medium amino acid supplements (Sigma–Aldrich, Steinheim, Germany), and 20 g/L agar] and incubated at 30°C for 2–3 days. Transformants were re-streaked at least three times on SC⁻^URA^ media to remove any background, and the cloned genes were confirmed with colony PCR. Positive transformants were cultivated in 15 mL SC⁻^URA^ broth (supplemented with 0.2 g/L chloramphenicol) at 30°C and 120 rpm for 3 days. Plasmid DNA was isolated with the Zyppy™ Plasmid Miniprep Kit (Zymo Research, CA, USA); the protocol was adjusted to include a 5-min bead-beater step (at maximum rpm) in 7X Lysis Buffer. After transformation in *E. coli* competent cells and propagation, the clones were confirmed with restriction digests and/or gene sequencing, and the yeast strains were re-transformed with the correct plasmids.

#### Co-transforming with plasmids pHV3 or YEplac181

Yeast fumarase transformants were co-transformed with plasmid pHV3 as described above. Plasmid pHV3 contains the *S. pombe mae1* open reading frame under the control of the *PGK1* promoter and terminator sequences (Volschenk et al. 1997b), as well as a leucine marker. Fumarase transformants were also co-transformed with YEplac181 to generate no-transporter control strains that were able to grow on SC⁻^URA^⁻^LEU^ media.

#### Screening of transformants

Standard curves for dry cell weight (DCW) versus absorbance at 600 nm (WPA Lightwave-II UV/Visible Spectrophotometer, Biochrom) were compiled for the Sc-FUM1⁻ and Y294 parental strains. Cell suspensions were vacuum-filtered through 47-mm glass microfiber filters (Whatman™, Maidstone, UK), oven-dried, and re-weighed. For the different cultivations (see below), 2 mL aliquots were collected in triplicate every 24 h, absorbance measured at 600 nm and transferred to microcentrifuge tubes with 50 μL of a 10% (v/v) H_2_SO_4_ solution and stored at −20°C. After thawing, the yeast cultures were centrifuged at 13 000 rpm for 10 min and the supernatants filter-sterilized into glass vials using 0.22-µm nylon syringe filters (Anatech, Randburg, South Africa). The l-malic acid, glucose, ethanol, glycerol, acetic acid, fumaric acid, lactic acid, and succinic acid concentrations in the supernatant were quantified with high-performance liquid chromatography (HPLC) on a Surveyor Plus liquid chromatograph (Thermo Fisher Scientific) consisting of an LC pump, autosampler, and refractive index detector. Samples were separated on the Rezex RHM monosaccharide polymer-based column (300 × 7.8 mm) at 80°C using a Gecko 2000 column heater using a 5 mM sulphuric acid mobile phase at a flow rate of 0.6 mL/min. Internal standards of known concentrations were included to assess the accuracy of the HPLC analyses. Results indicate the average value from triplicate cultures, and standard errors were used to determine significant differences between strains.

#### Small-scale cultivations

Small-scale cultivations were performed in 96-deep-well plates using SC⁻^URA^⁻^LEU^ broth (pH 5) with either 15 g/L l-malic acid plus 5 g/L glucose (ratio of 3:1) or 15 g/L l-malic acid plus 1 g/L glucose (ratio of 15:1) as carbon sources. The transformants were precultured overnight in their respective media and absorbance was measured at 600 nm (with path length corrected for the volume) using a BIO-RAD xMark™ Microplate Spectrophotometer (BIO-RAD, Hercules, CA, USA). The inoculum volume for each transformant was standardized to an A_600_ of 0.5 and the transformants were cultivated at 30°C for 72 h in 96-deep-well plates containing 2 mL of either the 3:1 or 15:1 cultivation media. The plates were covered with a sterile Breathe Easier sealing membrane (allowing CO_2_, O_2_, and water vapour to permeate) (Sigma–Aldrich, St. Louis, MI, USA) and a sterile 2-mm glass bead in each well facilitated mixing at 600 rpm. Triplicate samples were taken every 24 h and frozen for HPLC analyses.

#### Upscaled cultivation of selected strains under aerobic and oxygen-limited conditions

The ability of the recombinant strains to convert malic acid into fumaric acid was evaluated in SC⁻^URA^⁻^LEU^ media with a 10:1 malic acid:glucose ratio (11.11 g/L malic acid and 1.11 g/L glucose) under aerobic and oxygen-limited conditions. The Sc-FUM^−^ strains (*ΔFUM1Ckr_fum* + *mae1* and *ΔFUM1*(ss)*Ckr_fum* + *mae1*) and their Y294 counterparts (WT*Ckr_fum* + *mae1* and WT(ss)*Ckr_fum* + *mae1*) were cultivated in triplicate for 72 h in 10:1 SC⁻^URA^⁻^LEU^ broth under aerobic conditions (45 mL broth plus 5 mL inoculum) using 500 mL baffled Erlenmeyer flasks or under oxygen-limited conditions (90 mL broth plus 10 mL inoculum) in 100 mL serum bottles sealed with rubber stoppers. The pH of the cultures were monitored, and triplicate samples were collected every 24 h for HPLC and DCW (g/L) analyses.

## Results and discussion

Previous studies on biobased fumaric acid production have mainly relied on pure glucose, lignocellulosic biomass hydrolysate sugars, or starchy materials as substrates (Sebastian et al. 2021, Guo et al. 2020). The current study aimed to construct *S. cerevisiae* strains that can produce fumaric acid from extracellular malic acid. Preliminary data (not shown) illustrated weak malic acid metabolism and no fumaric acid production by the parental *S. cerevisiae* S288c and Y294 strains. To enable the conversion of malic to fumaric acid, recombinant strains were constructed to express a fumarase gene from either yeast (*Ckr_fum* with/without the XYNSEC secretion signal) or bacterial origin (*Eco_fum* with the XYNSEC secretion signal), together with the *S. pombe* malate transporter gene (*mae1*). No-transporter control strains (without *mae1*) were co-transformed with YEplac181 to provide a leucine marker for cultivation in SC⁻^URA^⁻^LEU^ media. The recombinant strains were compared in terms of the respective fumarase and transporter genes in the Sc-FUM1⁻ (*ΔFUM1*) versus Y294 (WT) strains to assess the impact of the disrupted native *S. cerevisiae FUM1* gene. The strains were evaluated in different glucose and malic acid concentrations for malic acid utilization and fumaric acid production, among other parameters. As the utilization of malic acid and production of other organic acids are mainly associated with the TCA cycle, all the cultivations were performed under aerobic conditions, with selected upscaled cultivations performed in oxygen-limited conditions for comparative purposes.

### Constructing recombinant strains

A total of 18 yeast strains with different combinations of fumarases (with/without a secretion signal) and with/without a transporter (+*mae1* or +*LEU2*) were constructed (Table 3). Cloning the bacterial *Eco_fum* gene in pBBH1 (no secretion signal) proved problematic and was not pursued further. Amplification of the *Ckr_fum* and *Eco_fum* genes produced fragments of 1470 bp and 1416 bp, respectively, whose integrity was confirmed with sequencing alignments (data not shown). Restriction digests and YML of the fumarase genes yielded plasmids pBBH1-Ckr_fum (7765 bp), pBBH4-Ckr_fum (7889 bp), and pBBH4-Eco_fum (7835 bp), which were successfully transformed into the Sc-FUM1⁻ and Y294 strains. These strains were successfully co-transformed with either plasmid pHV3 (with *mae1* transporter gene) or plasmid YEplac181 (no-transporter control strains).

**Table 3.** Recombinant strains expressing a foreign fumarase with or without the XYNSEC secretion signal (ss) and co-transformed with plasmids pHV3 (expressing *mae1*) or YEplac181 (no-transporter control).

Parental strains	XYNSEC secretion signal (ss)^a^	+ fumarase^b^	+ fumarase + pHV3^c^	+ fumarase + YEplac181^c^
*S. cerevisiae FUM1^−^* (Y11030) *→ ∆FUM1*	− + +	*∆FUM1Ckr_fum* *∆FUM1*(ss)*Ckr_fum* *∆FUM1*(ss)*Eco_fum*	*∆FUM1Ckr_fum* +* mae1* *∆FUM1*(ss)*Ckr_fum* +* mae1* *∆FUM1*(ss)*Eco_fum* +* mae1*	*∆FUM1Ckr_fum* +* LEU2* *∆FUM1*(ss)*Ckr_fum* +* LEU2* *∆FUM1*(ss)*Eco_fum* +* LEU2*
*S. cerevisiae* Y294 (ATCC 201160) *→* WT	− + +	WT*Ckr_fum* WT(ss)*Ckr_fum* WT(ss)*Eco_fum*	WT*Ckr_fum* +* mae1* WT(ss)*Ckr_fum* +* mae1* WT(ss)*Eco_fum* +* mae1*	WT*Ckr_fum* +* LEU2* WT(ss)*Ckr_fum* +* LEU2* WT(ss)*Eco_fum* +* LEU2*

a Cloned in pBBH1 (−) or pBBH4 (+).

b Transformants selected on SC^−URA^.

c Transformants selected on SC^−URA-LEU^.

### Small-scale cultivations with adjusted malic acid:glucose ratios

#### *Saccharomyces cerevisiae* FUM1⁻ transformants

Overflow metabolism (the Crabtree effect) is a well-known phenomenon in *S. cerevisiae* that results in the production of glycerol and ethanol under aerobic conditions in the presence of high glucose concentrations (Vemuri et al. 2007). To avoid excess glycerol production, l-malic acid:glucose ratios of either 3:1 or 15:1 were used to evaluate the new strains. Note that precultures may have introduced some additional glucose and malic acid to the cultures, as well as low levels of ethanol from the ethanol-dissolved chloramphenicol and/or due to ethanol production in some precultures. Substrate not converted into products likely contributed to biomass formation. The performance of selected Sc-FUM1⁻ strains, *ΔFUM1*(ss)*Ckr_fum* + *mae1* and *ΔFUM1*(ss)*Eco_fum* + *mae1*, was compared to their no-transporter control strains.

**Figure 1.**
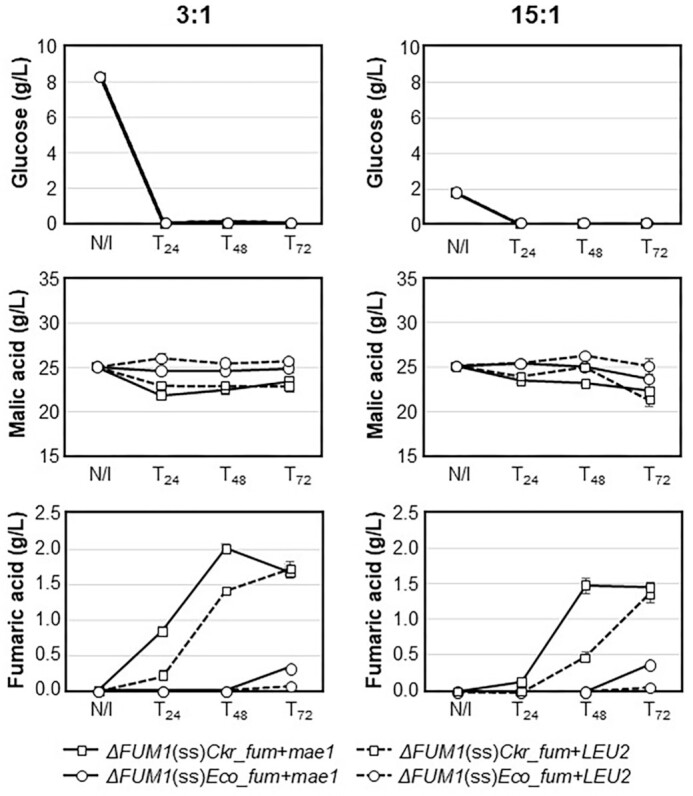
Residual glucose, malic acid, and fumaric acid concentrations following cultivation of Sc-FUM1^–^ transformants in 96-deep-well plates using SC^–URA–LEU^ broth with either 3:1 (left) or 15:1 (right) ratios of malic acid:glucose. Results represent the averages from triplicate cultures of strains *ΔFUM1*(ss)*Ckr_fum* + *mae1* (squares; solid lines) and *ΔFUM1*(ss)*Eco_fum* +* mae1* (circles; solid lines), as well as their no-transporter controls, *ΔFUM1*(ss)*Ckr_fum* + *LEU2* and *ΔFUM1*(ss)*Eco_fum* +* LEU2* (dashed lines). N/I = no inocula. Error bars indicate standard errors.

As shown in Fig. 1, all the strains depleted the glucose in the growth media within the first 24 h, whereas the malic acid degradation profiles varied. The K(−) yeasts (such as *S. cerevisiae* and *S. pombe*) can utilize TCA cycle intermediates only in the presence of glucose or other assimilable carbon source and thus requires glucose to use extracellular malic acid (Volschenk et al. 2003). In the 3:1 (malic acid:glucose) media, the transporter-carrying strains (+*mae1*) degraded more malic acid than the control strains (+*LEU2*) over the first 24 h, thus confirming that SpMae1p contributed towards the initial uptake of malic acid. The *ΔFUM1*(ss)*Ckr_fum* + *mae1* strain (yeast fumarase) utilized 12.79% of the extracellular malic acid at T_24_, whereas *ΔFUM1*(ss)*Eco_fum* + *mae1* (bacterial fumarase) only utilized 1.94%. In the 15:1 media with less glucose, strain *ΔFUM1*(ss)*Ckr_fum* + *mae1* was the best candidate after 48 h, using 6.47% of the extracellular malic acid. Not all the malic acid consumed was converted into fumaric acid since *S. cerevisiae* cells can use TCA cycle intermediates for biomass and other metabolic requirements. Furthermore, significant levels of malic acid remained unutilized, highlighting the need for a recombinant fumarase.

A more significant difference was evident for the extracellular fumaric acid concentrations. In the 3:1 medium, strain *ΔFUM1*(ss)*Ckr_fum* + *mae1* produced 2.01 g/L fumaric acid after 48 h, which was significantly higher than its counterpart without Spmae1 (1.49 g/L), although the two strains achieved similar levels at T_72_ (Fig. 1). The same trend was observed in the 15:1 medium, with *ΔFUM1*(ss)*Ckr_fum* + *mae1* producing 1.49 g/L fumaric acid at T_48_ versus 0.49 g/L by *ΔFUM1*(ss)*Ckr_fum* + *LEU2*. In both media, the strains expressing the bacterial fumarase, *ΔFUM1*(ss)*Eco_fum* + *mae1* and *ΔFUM1*(ss)*Eco_fum* + *LEU2*, produced significantly less fumaric acid than strains expressing the yeast fumarase. The presence of Spmae1p increased the extracellular levels of fumaric acid when used in combination with the yeast or bacterial fumarase, but with a more significant impact for the yeast fumarase.

Since the fumarase gene from *C. kruseii* (Finley et al. 2013) was more effective in fumaric acid production, the *Eco_fum*-based strains were excluded from subsequent experiments.

#### *Saccharomyces cerevisiae* Y294 transformants

**Figure 2.**
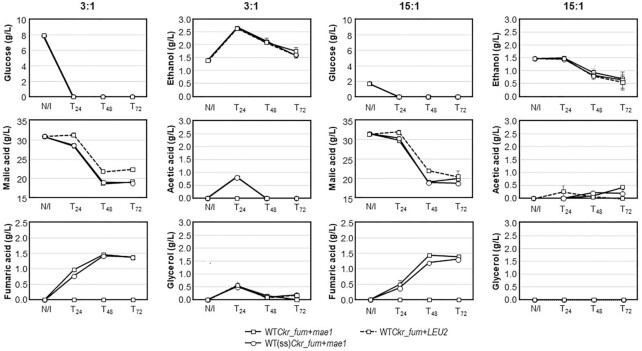
Residual glucose, malic acid, fumaric acid, ethanol, acetic acid, and glycerol concentrations following cultivation of Y294 transformants in 96-deep-well plates using SC^–URA–LEU^ broth with either 3:1 (left) or 15:1 (right) ratios of malic acid:glucose. Results represent the averages from triplicate cultures of strains WT*Ckr_fum* +* mae1* (squares; solid lines) and WT(ss)*Ckr_fum* +* mae1* (circles; solid lines) as well as the no-transporter control, WT*Ckr_fum* +* LEU2* (dashed lines). N/I = no inocula. Error bars indicate standard errors.

The performance of selected Y294 transformants was also investigated to determine the effect of the intact *FUM1* gene that favours fumarate-to-malate conversion in yeasts. All the strains, namely WT*Ckr_fum* + *mae1*, WT*Ckr_fum* + *LEU2*, and WT(ss)*Ckr_fum* + *mae1*, depleted the glucose within the first 24 h in both carbon source ratios (Fig.   2). Strains WT*Ckr_fum* + *mae1* and WT(ss)*Ckr_fum* + *mae1* outperformed their no-transporter counterpart in terms of malic acid utilization and fumaric acid production, although strain WT*Ckr_fum* + *mae1* produced slightly more fumaric acid at T_48_ in the 15:1 carbon ratio. The glucose content did not impact fumaric acid production, with strain WT*Ckr_fum* + *mae1* reaching 1.47 and 1.44 g/L after 48 h, in the 3:1 and 15:1 carbon ratios, respectively. The no-transporter control strain (WT*Ckr_fum* + *LEU2*) produced no fumaric acid during cultivation in either media, illustrating the advantage provided by the SpMae1p transporter.

In terms of the other by-products (Fig. 2), all the strains produced close to 0.5 g/L glycerol at T_24_ in the 3:1 media, after which glycerol levels decreased. The 15:1 medium resulted in varying levels of acetic acid (0.06–0.4 g/L), whilst the 3:1 media allowed for 0.71 g/L acetic acid production at 24 h for both WT*Ckr_fum* + *mae1* and WT(ss)*Ckr_fum* + *mae1*, but none for WT*Ckr_fum* + *LEU2*. All the strains produced maximum ethanol levels at T_24_ in the 3:1 media (1.24–1.26 g/L), but some of the ethanol was consumed over the following 48 h. No additional ethanol production was detected in the 15:1 media, with the ethanol levels decreasing after 24 h. The general trend of an initial increase and subsequent decrease in by-product levels could be explained by the initial glucose utilization (resulting in by-product formation) and the utilization of by-product carbon sources once glucose was no longer available.

### Upscaled comparisons of selected strains

**Figure 3.**
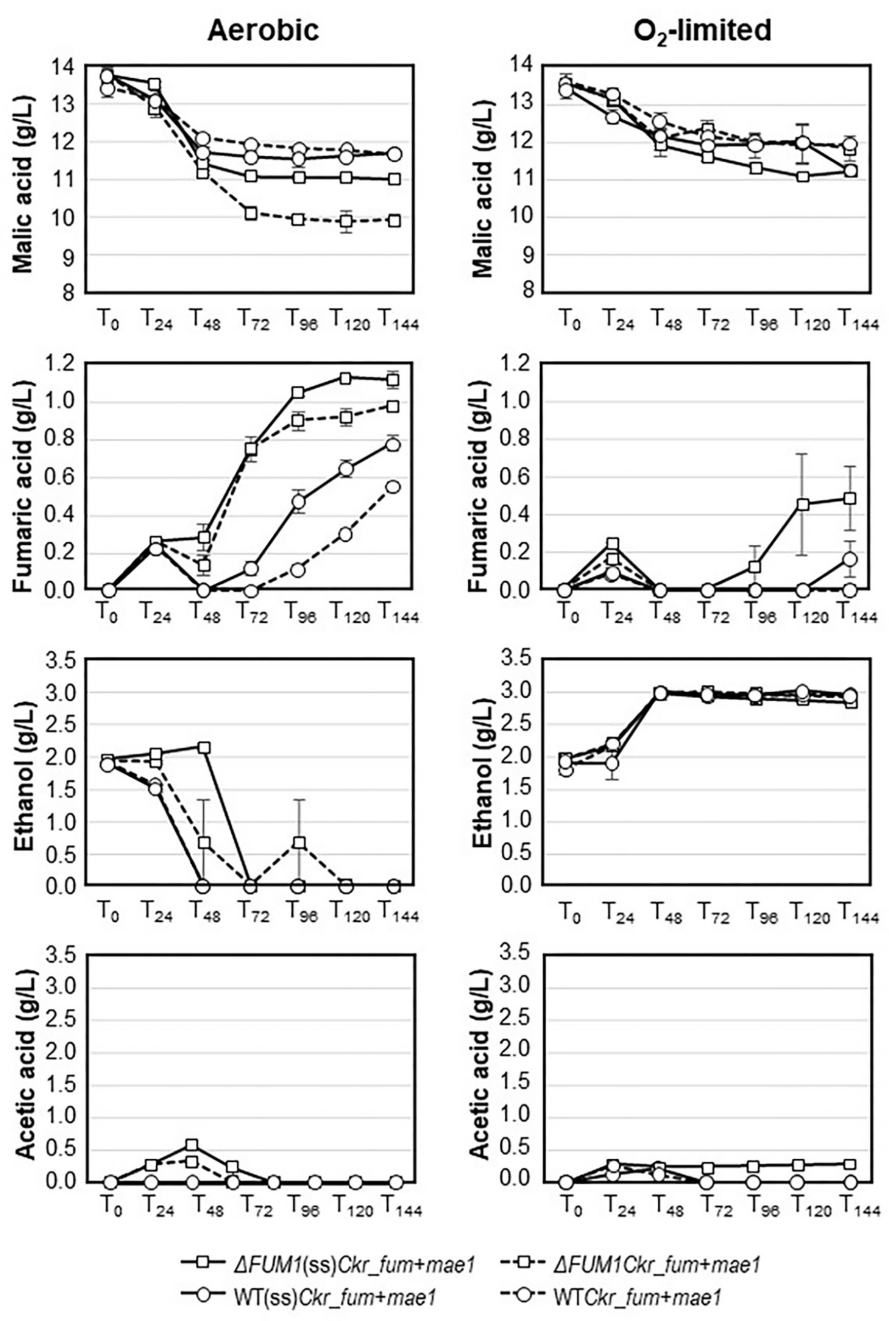
Residual malic acid and production of fumaric acid, ethanol, and acetic acid by selected *∆FUM*(ss)*Ckr_fum* +* mae1* (squares; solid lines) and WT(ss)*Ckr_fum* +* mae1* (circles; solid lines) strains relative to their counterparts without the XYNSEC secretion signal (dashed lines) under aerobic (left) and oxygen-limited (right) conditions following cultivation in SC^–URA–LEU^ media with a 10:1 ratio of malic acid to glucose. Data points represent the average of triplicate cultures, with error bars indicating standard errors.

The ability to convert malic acid into fumaric acid by the Sc-FUM⁻ strains relative to that of the Y294 strains was evaluated in upscaled cultivations using 10:1 SC⁻^URA^⁻^LEU^ media under aerobic and oxygen-limited conditions (Fig. 3). All strains depleted the available glucose within the first 24 h of cultivation under both oxygen parameters (data not shown). Based on DCW data (not shown), strains *ΔFUM1Ckr_fum* + *mae1* and *ΔFUM1*(ss)*Ckr_fum* + *mae1* grew similarly and were different from the Y294-based WT*Ckr_fum* + *mae1* and WT(ss)*Ckr_fum* + *mae1* strains, which grouped together.

Under aerobic conditions, strain *ΔFUM1Ckr_fum* + *mae1* was the best performer, with 26.6% of the extracellular malic acid utilized at T_72_. In contrast, its Y294 counterpart only used 11.2% (Fig. 3). Strain *ΔFUM1*(ss)*Ckr_fum* + *mae1* removed 19.7% of the malic acid, whilst its WT(ss)*Ckr_fum* + *mae1* Y294 counterpart only removed 13.6%. Both the Sc-FUM1⁻ strains also showed significantly higher levels of fumaric acid production at T_72_ than their Y294 counterparts. Fumaric acid production generally continued until T_144_ with malic acid conversion levels of 8.1% by *ΔFUM1*(ss)*Ckr_fum* + *mae1*, 5.8% by WT(ss)*Ckr_fum* + *mae1*, 7.1% by *ΔFUM1Ckr_fum* + *mae1*, and 4.1% by WT*Ckr_fum* + *mae1*. This supported our hypothesis that disruption of the irreversible *FUM1* gene in *S. cerevisiae* would benefit the production of fumaric acid from extracellular malic acid. The results also clearly show that fumaric acid production follows malic utilization, but both reached a plateau at 96 h when the glucose-depleted K(−) yeast strains could no longer sustain malic acid utilization.

Less malic acid was utilized under oxygen-limited conditions than under aerobic conditions, and all the strains produced less fumaric acid. However, *ΔFUM1*(ss)*Ckr_fum* + *mae1* produced slightly more fumaric acid than the other strains towards the end of the fermentation. Small amounts of acetic acid were detected up to 48 h; this coincided with an increase in ethanol at T_48_, after which ethanol levels remained steady at 2.8–2.9 g/L (Fig. 3). In contrast, ethanol levels decreased and were eventually depleted for all the aerobic cultivations.

Interestingly, the pBBH4 strains (secretion signal upstream of *fum*) generally showed better fumaric acid production than their counterparts without a secretion signal, which indicate possible extracellular fumaric acid production. The slightly better fumaric acid production by *ΔFUM1*(ss)*Ckr_fum* + *mae1* at 120 h in oxygen-limited conditions when the TCA cycle would be impeded, suggests that the XYNSEC secretion signal may have indeed helped with extracellular fumarase production. However, further investigation into the intracellular versus extracellular fumarase activity is required to confirm this.

## Conclusions

The current study aimed to construct *S. cerevisiae* strains that can produce fumaric acid from extracellular malic acid. Strain *ΔFUM1*(ss)*Ckr_fum* + *mae1* produced  1.49 and 2.01 g/L fumaric acid during small-scale cultivations on 15:1 and 3:1 malic acid:glucose media, respectively. While higher glucose concentrations resulted in more by-product formation (such as glycerol and ethanol), the addition of a transporter allowed for better malic-to-fumaric acid conversion. The *ΔFUM1Ckr_fum* + *mae1* and *ΔFUM1*(ss)*Ckr_fum* + *mae1* strains, respectively, produced 0.98 g/L and 1.11 g/L fumaric acid (aerobic) and 0.17 g/L and 0.49 g/L fumaric acid (O _2_-limited) in upscaled cultivations (10:1 malic acid:glucose) at T_144_, suggesting that the XYNSEC secretion signal allowed for better fumaric acid production. Furthermore, disruption of the natural *FUM1* gene in *S. cerevisiae* strains proved beneficial when using malic acid as a substrate for fumaric acid production.

## References

[bib1] Camarasa C , Bidard F, Bony M et al. Characterization of *Schizosaccharomyces pombe* malate permease by expression in *Saccharomyces cerevisiae*. Appl Environ Microbiol. 2001;67:4144–51. 11526017 10.1128/AEM.67.9.4144-4151.2001 PMC93141

[bib2] ChemAnalyst . Fumaric Acid Price Trend and Forecast. 2022. https://www.chemanalyst.com/Pricing-data/fumaric-acid-1134 (9 December 2022, date last accessed).

[bib3] Chen X , Zhu P, Liu L. Modular optimization of multi-gene pathways for fumarate production. Metab Eng. 2016;33:76–85. 26241189 10.1016/j.ymben.2015.07.007

[bib4] Cripwell RA , Rose SH, Favaro L et al. Construction of industrial *Saccharomyces cerevisiae* strains for the efficient consolidated bioprocessing of raw starch. Biotechnol Biofuels. 2019;12:201. 31452682 10.1186/s13068-019-1541-5 PMC6701143

[bib5] Darbani B , Stovicek V, Van Der Hoek SA et al. Engineering energetically efficient transport of dicarboxylic acids in yeast *Saccharomyces cerevisiae*. Proc Natl Acad Sci. 2019;116:19415–20. 31467169 10.1073/pnas.1900287116 PMC6765260

[bib21] Del Sal G , Manfioletti G, Schneider C. A one-tube plasmid DNA mini-preparation suitable for sequencing. Nucleic Acids Res. 1988;16:9878. 3186460 10.1093/nar/16.20.9878 PMC338806

[bib6] Engel C , Straathof AJJ, Zijlmans TW et al. Fumaric acid production by fermentation. Appl Microbiol Biotechnol. 2008;78:379–89. 18214471 10.1007/s00253-007-1341-x PMC2243254

[bib7] Finley KR , Huryta JM, Mastel BM et al. Compositions and methods for succinate production. US patent US 9,605,285 B2 2017. DOI: 10.4161/bbug.2.2.14549.

[bib37_1674133721661] Gietz RD , Sugino A. A. New yeast-*Escherichia coli* shuttle vectors constructed with in vitro mutagenized yeast genes lacking six-base pair restriction sites. Gene. 1988;74:527–34. 3073106 10.1016/0378-1119(88)90185-0

[bib8] Grobler J , Bauer F, Subden RE et al. The *mae1* gene of *Schizosaccharomyces pombe* encodes a permease for malate and other C4 dicarboxylic acids. Yeast. 1995;11:1485–91. 8750236 10.1002/yea.320111503

[bib9] Gu S , Li J, Chen B et al. Metabolic engineering of the thermophilic filamentous fungus *Myceliophthora thermophila* to produce fumaric acid. Biotechnol Biofuels. 2018;11:1–10. 30534201 10.1186/s13068-018-1319-1 PMC6278111

[bib10] Guo F , Wu M, Dai Z et al. Current advances on biological production of fumaric acid. Biochem Eng J. 2020;153:107397.

[bib11] Husnik JI , Volschenk H, Bauer J et al. Metabolic engineering of malolactic wine yeast. Metab Eng. 2006;8:315–23. 16621641 10.1016/j.ymben.2006.02.003

[bib12] Ichikawa S , Iino T, Sato S et al. Improvement of production rate and yield of fumaric acid from maleic acid by heat treatment of *Pseudomonas alcaligenes* strain XD-1. Biochem Eng J. 2003;13:7–13.

[bib13] Ilica RA , Kloetzer L, Galaction AI et al. Fumaric acid: production and separation. Biotechnol Lett. 2019;41:47–57. 30506453 10.1007/s10529-018-2628-y

[bib14] Laemmli UK . Cleavage of structural proteins during the assembly of the head of bacteriophage T4. Nature. 1970;227:680–5. 5432063 10.1038/227680a0

[bib15] Nevoigt E . Progress in metabolic engineering of *Saccharomyces cerevisiae*. Microbiol Mol Biol Rev. 2008;72:379–412. 18772282 10.1128/MMBR.00025-07 PMC2546860

[bib16] Njokweni AP , Rose SH, Van Zyl WH. Fungal β-glucosidase expression in *Saccharomyces cerevisiae*. J Ind Microbiol Biotechnol. 2012;39:1445–52. 22707073 10.1007/s10295-012-1150-9

[bib17] Njokweni SG , Steyn A, Botes M et al. Potential valorization of organic waste streams to valuable organic acids through microbial conversion: a South African case study. Catalysts. 2021;11:964.

[bib18] Pines O , Even-Ram S, Elnathan N et al. The cytosolic pathway of L-malic acid synthesis in *Saccharomyces cerevisiae*: the role of fumarase. Appl Microbiol Biotechnol. 1996;46:393–9. 8987728 10.1007/BF00166235

[bib19] Raab AM , Gebhardt G, Bolotina N et al. Metabolic engineering of *Saccharomyces cerevisiae* for the biotechnological production of succinic acid. Metab Eng. 2010;12:518–25. 20854924 10.1016/j.ymben.2010.08.005

[bib20] Saayman M , Viljoen-Bloom M. The biochemistry of malic acid metabolism by wine yeasts—a review. South African J Enol Vitic. 2006;27:113–22.

[bib22] Sambrook J , Fritsch E, Maniatis T. Molecular Cloning: A Laboratory Manual, Vol. 9. Cold Spring Harbor, NY: Cold Spring Harbor Laboratory Press, 1989.

[bib23] Sebastian J , Dominguez KV, Brar SK et al. Fumaric acid production using alternate fermentation mode by immobilized *Rhizopus oryzae*—a greener production strategy. Chemosphere. 2021;281:130858. 34020187 10.1016/j.chemosphere.2021.130858

[bib24] Sousa MJ , Mota M, Leão C. Transport of malic acid in the yeast *Schizosaccharomyces pombe*: evidence for proton-dicarboxylate symport. Yeast. 1992;8:1025–31. 1293882 10.1002/yea.320081205

[bib25] Steyn A , Viljoen-Bloom M, van Zyl WH. Valorization of apple and grape wastes with malic acid-degrading yeasts. Folia Microbiol (Praha). 2021;66:341–54. 33474701 10.1007/s12223-021-00850-8

[bib26] Vemuri GN , Eiteman MA, McEwen JE et al. Increasing NADH oxidation reduces overflow metabolism in *Saccharomyces cerevisiae*. Proc Natl Acad Sci USA. 2007;104:2402–7. 17287356 10.1073/pnas.0607469104 PMC1892921

[bib29] Volschenk H , van Vuuren HJJ, Viljoen-Bloom M. Malo-ethanolic fermentation in *Saccharomyces* and *Schizosaccharomyces*. Curr Genet. 2003;43:379–91. 12802505 10.1007/s00294-003-0411-6

[bib28] Volschenk H , Viljoen M, Grobler J et al. Engineering pathways for malate degradation in *Saccharomyces cerevisiae*. Nat Biotechnol. 1997b;15:253–7. 9062925 10.1038/nbt0397-253

[bib27] Volschenk H , Viljoen M, Grobler J et al. Malolactic fermentation in grape musts by a genetically engineered strain of *Saccharomyces cerevisiae*. Am J Enol Vitic. 1997a;48:193–7.

[bib30] Wei L , Liu J, Qi H et al. Engineering *Scheffersomyces stipitis* for fumaric acid production from xylose. Bioresour Technol. 2015;187:246–54. 25863201 10.1016/j.biortech.2015.03.122

[bib31] Werpy T , Petersen G (eds). Results of Screening for Potential Candidates from Sugars and Synthesis Gas, Top Value Added Chemicals from Biomass, Vol. I, NREL, Oak Ridge, TN, USA: U.S. Department of Energy, 2004, 1–76.

[bib32] Xu G , Chen X, Liu L et al. Fumaric acid production in *Saccharomyces cerevisiae* by simultaneous use of oxidative and reductive routes. Bioresour Technol. 2013;148:91–6. 24045196 10.1016/j.biortech.2013.08.115

[bib33] Xu G , Liu L, Chen J. Reconstruction of cytosolic fumaric acid biosynthetic pathways in *Saccharomyces cerevisiae*. Microb Cell Fact. 2012;11:24. 22335940 10.1186/1475-2859-11-24 PMC3340314

[bib34] Xu G , Shi X, Gao Y et al. Semi-rational evolution of pyruvate carboxylase from *Rhizopus oryzae* for elevated fumaric acid synthesis in *Saccharomyces cerevisiae*. Biochem Eng J. 2022;177:108238.

[bib35] Yin X , Li J, Shin H et al. Metabolic engineering in the biotechnological production of organic acids in the tricarboxylic acid cycle of microorganisms: advances and prospects. Biotechnol Adv. 2015;33:830–41. 25902192 10.1016/j.biotechadv.2015.04.006

[bib36] Zelle RM , Hulster DE, Winden VWA et al. Malic acid production by *Saccharomyces cerevisiae*: engineering of pyruvate carboxylation, oxaloacetate reduction, and malate export. Appl Environ Microbiol. 2008;74:2766–77. 18344340 10.1128/AEM.02591-07 PMC2394876

